# Yishen-Huoxue formula alleviates renal interstitial fibrosis by attenuating hypoxia-induced renal cell injury and promoting angiogenesis via miR-210/HIF-1α pathway

**DOI:** 10.3389/fmed.2025.1530092

**Published:** 2025-05-21

**Authors:** Mouying Du, Chun Yao, Qinke Lv, Aimei Gong, Yonghua Zhu, Jiayuan Li, Jian Zhong

**Affiliations:** ^1^Graduate School, Guangxi University of Traditional Chinese Medicine, Nanning, China; ^2^Guangxi University of Traditional Chinese Medicine, Nanning, China; ^3^Department of Nephrology, The First Affiliated Hospital of Guangxi University of Traditional Chinese Medicine, Nanning, China

**Keywords:** renal interstitial fibrosis, Yishen-Huoxue formula, miR-210, HIF-1α, hypoxia

## Abstract

**Background:**

Renal interstitial fibrosis (RIF) represents the final common pathway in nearly all progressive chronic kidney diseases. This study aimed to investigate the effects of Yishen-Huoxue formula (YHF) on RIF and its underlying mechanisms.

**Methods:**

Unilateral ureteral obstruction (UUO) model was applied to induce RIF *in vivo*. Thirty-six male SD rats were randomized into six groups: sham group, UUO group, UUO + Losartan group, and UUO + YHF-L/M/H groups. The histopathological changes of rat kidney and its function were evaluated 14 days post-UUO. miRNA sequencing and differential gene analysis identified miR-210 as a potential target of YHF treatment. Western blot and qRT-PCR were employed to assess the impact of miR-210 overexpression and knockdown on the expression of hypoxia-related factors in HK-2, NRK-52E, HUVEC, and 293T cells under hypoxic conditions. Cell proliferation, migration, and angiogenesis were determined using CCK-8 assays, transwell assays, and tubule formation assays, respectively.

**Results:**

*In vivo*, YHF treatment significantly attenuated RIF, protected renal function, increased miR-210 expression, and decreased the expression of HIF-1α, BNIP3, and NIX in the serum exosomes of UUO rats. *In vitro* experiments revealed that miR-210 downregulates the expression of HIF-1α and its downstream factors, mitigating hypoxia-induced cellular injuries, including decreased cell viability, migration ability, and angiogenesis capability. Further experiments demonstrated that YHF treatment upregulated miR-210 expression while downregulating HIF-1α expression in HK-2 cells under hypoxic conditions. Meanwhile, YHF alleviated hypoxia-induced renal cell damage and impaired angiogenesis in HUVECs, with miR-210 mimic enhancing and miR-210 inhibitor attenuating these protective effects.

**Conclusion:**

Yishen-Huoxue formula alleviates RIF by mediating the miR-210/HIF-1α pathway to attenuate hypoxia-induced renal cell injury and promote angiogenesis.

## 1 Introduction

Renal interstitial fibrosis (RIF) is the final common pathway leading to end-stage renal disease in nearly all progressive chronic kidney diseases (CKD) and serves as a reliable indicator of patient prognosis (1). Therefore, alleviating RIF is crucial for improving disease outcomes. Hypoxia/ischemia serves as a significant contributor to the onset and progression of RIF (2). The tubular epithelial cell (TEC) hypoxia and peritubular capillary (PTC) rarefaction are recognized as important features of RIF (3, 4). Studies have shown that the loss of PTC disrupts the delivery of oxygen and nutrients, leading to renal tubular and interstitial injury, which in turn stimulates the development of RIF. Moreover, RIF and the accumulation of extracellular matrix further exacerbate the occlusion of PTC, expanding the hypoxic/ischemic regions, thereby creating a vicious cycle that promotes RIF (5, 6). Consequently, mitigating TEC injury induced by hypoxia and promoting renal microvascular regeneration are highly desirable strategies to alleviate RIF.

Yishen-Huoxue formula (YHF) is a modified prescription based on traditional Chinese medicine principles, composed of six herbs—Astragalus, Angelica, Safflower, Salvia, Rhubarb, and Notoginseng—with the effects of tonifying the kidney, promoting blood circulation, and replenishing Qi. In recent years, this formula has been used to treat CKD and its complications, demonstrating positive therapeutic effects in alleviating RIF (7, 8). Our previous studies have shown that YHF ameliorates endogenous hypoxia by regulating hypoxia-inducible factors like HIF-1α in the unilateral ureteral obstruction (UUO) rat model (9). Additionally, we found that YHF can upregulate vascular endothelial growth factor via miR-126, promoting the repair of PTC damage in RIF (10). Thus, YHF may exert anti-RIF effects by effectively intervening in the hypoxia and angiogenesis of renal cells. However, we found in in vitro experiments that even when miR-126 was knocked down in human umbilical vein endothelial cells (HUVEC) cultured under hypoxia, YHF treatment still promoted the formation of new blood vessels on Matrigel. This indicated that miR-126 is not the sole factor required for angiogenesis in vitro and that other upstream regulatory factors of endothelial cell (EC) proliferation may exist.

In recent years, the regulatory role of miR-210 in hypoxia and angiogenesis has garnered significant attention. Research by Zhao et al. demonstrated that hypoxic stimulation *in vitro* upregulates miR-210 expression in HUVEC and promotes angiogenesis (11). Recent studies have also shown that in hypoxic kidney disease models, miR-210 targets HIF-1α and inhibits the activation of downstream target genes of HIF-1α following severe hypoxia, mitigating hypoxia-induced apoptosis (12). These findings suggest that miR-210 may be a potential target for the treatment of kidney disease. Given the close correlation between miR-210 and HIF-1α with hypoxia and angiogenesis, we hypothesized that YHF attenuates renal cell injury and promotes angiogenesis under hypoxic conditions by mediating factors such as miR-210 and HIF-1α, thereby alleviating RIF.

## 2 Materials and methods

### 2.1 Animals

A total of 36 SFP-grade male Sprague-Dawley (SD) rats, weighing 180–200 g, were obtained from the Experimental Center of Guangxi University of Traditional Chinese Medicine (Nanning, China, Certificate No. SCXK (Gui) 2020-0003). All experimental procedures involving animals were conducted in strict accordance with ethical guidelines and were approved by the Ethics Committee of Guangxi University of Traditional Chinese Medicine (Approval No. DW20240614-178).

### 2.2 Medicine preparation

The composition of YHF includes 15 g of Astragalus [*Astragalus membranaceus* (Fisch.) Bunge], 10 g of Angelica [*Angelica sinensis* (Oliv.) Diels], 15 g of Safflower (*Carthamus tinctorius* L.), 15 g of Salvia (*Salvia miltiorrhiza* Bunge), 8 g of Rhubarb (*Rheum palmatum* L.), and 6 g of Notoginseng [*Panax notoginseng* (Burkill) F. H.Chen] (7, 8). The ready-to-use YHF granules (containing all six herbal components in the specified ratio) were purchased from Jiangyin Tianjiang Pharmaceutical Co., Ltd. (Jiangyin, China) and required no further decoction. Before use, the YHF granules were fully dissolved in distilled water and diluted to a concentration of 1.18 g/mL. Losartan (brand name: Cozaar, H20000371) was purchased from Hangzhou MSD Pharmaceutical Co., Ltd., with each tablet containing 50 mg. The tablets were ground into powder and prepared into a 12.5 mg/mL solution using distilled water.

### 2.3 Animal grouping

Thirty-six rats were divided into six groups according to the random number table method: the sham operation (sham) group, the UUO group, the UUO + Losartan treatment (UUO + LOS) group, and the UUO + low/medium/high dose YHF treatment (UUO + YHF-L/M/H) group. The five groups excluding the UUO + LOS group were utilized for in vivo experiments at the end of drug treatment [our previous research has established that YHF possesses comparable or superior RIF-mitigating effects to Losartan (10)]. Additionally, serum samples were collected from the sham, UUO, UUO + LOS, and UUO + YHF-L groups post-treatment for the purposes of sequencing and differential gene analysis.

The rat model of RIF was induced through the UUO (13). The surgical method was as follows: The rats were fixed in supine position on an operating table after intraperitoneal anesthesia with 5% pentobarbital sodium (50 mg/kg). Then routine sterilization was performed. The left ureter was ligated proximally with a 4–0 silk thread in two passes and cut between the ligations. And the sham group was operated in the same way, while the ureter was not ligated and disconnected.

The method of administration was as follows: For the UUO + YHF-H/M/L groups, rats were given YHF by gavage at doses of 28.4, 14.2, and 7.1 g/kg once a day in the morning, respectively, starting on the day modeling was completed. The required doses of YHF were calculated based on the equivalent dose conversion formula for humans and rats, corresponding to 2, 1, and 0.5 times the clinical equivalent dose, respectively. The UUO + LOS group received 50 mg/kg of Losartan by gavage once daily in the morning, starting on the day modeling was completed. The UUO group and sham group were given an equivalent volume of physiological saline, administered once daily. The drug interventions continued until the day the rats were sacrificed. All rats were sacrificed on the 14th day post-surgery, and kidney tissues were collected, along with blood samples, which were centrifuged to obtain the supernatant.

### 2.4 Masson trichrome staining

Kidney tissues of rats were fixed in 4% paraformaldehyde at 4°C for 24 h. The tissues were then dehydrated with graded ethanol and embedded in paraffin. The embedded kidney tissues were sectioned into 4 μm thick slices and stained with Masson’s trichrome stain (Solaribio, G1340) to assess pathological changes (14).

### 2.5 Immunohistochemistry (IHC)

Kidney tissue sections of rats were deparaffinized and rehydrated. Antigen retrieval was performed using EDTA antigen retrieval solution. The sections were then blocked with 10% goat serum blocking solution. Primary antibodies (α-Smooth Muscle Actin (D4K9N) XP^®^ Rabbit mAb #19245 from CST and Anti-Collagen IV antibody ab6586 from Abcam) were applied to the sections and incubated overnight at 4°C. Subsequently, secondary antibody [SignalStain^®^ Boost IHC Detection Reagent (HRP, Rabbit)] was added. All sections were stained with 3,3′-diaminobenzidine solution and counterstained with hematoxylin (15). After dehydration with graded alcohol and mounting with resin, the sections were examined and photographed under a light microscope.

### 2.6 Renal function tests

Blood samples of rats were collected. After centrifugation at 1,000 rpm for 5 min at 4°C, the serum samples were cryopreserved at −*80*°C. Serum uric acid (UA) levels were detected using the UA enzyme-linked immunosorbent assay kit (Huabang BIO, China) according to the manufacturer’s instructions. The levels of serum creatinine (Scr) and blood urea nitrogen (BUN) were measured using a Beckman AU480 chemistry analyzer (Beckman Coulter Inc., United States) (16, 17).

### 2.7 Serum exosomes isolation and RNA extraction

Rat drug-containing serum (1 mL) was centrifuged at 2,000 × *g* for 10 min at 4°C, and then the supernatant was collected and centrifuged at 10,000 × *g* for 20 min at 4°C (18). After collecting the supernatant, exosomes were extracted using the ExoQuick-TC PLUS Exosome Extraction Kit (SBI System Biosciences, United States) according to the manufacturer’s protocol. Total RNA was extracted from exosomes using TRIzol^®^ reagent (Invitrogen, United States) according to the manufacturer’s instructions.

### 2.8 Differential expression analysis of exosome miRNA

Unique molecular identifier (UMI) small RNA sequencing of rat exosomal microRNAs was performed to screen for expression of microRNAs associated with hypoxia/angiogenesis. Differential expression of miRNAs was determined using a criteria of *p*-value < 0.05 & |log_2_ FC| ≥ 1 (19). Cytoscape^1^ was employed for visualizing the miRNA-mRNA network and describing the results. Gene Ontology (GO^2^) and DAVID 6.8^3^ were used for GO and KEGG pathway analysis, with a threshold set at *p* < 0.05 (20).

### 2.9 Cell culture and treatment

All cell lines [human kidney-2 (HK-2) cells, human embryonic kidney 293T (293T), rat renal tubular epithelium cells (NRK-52E), and HUVEC] were obtained from the Cell Bank of the Chinese Academy of Sciences (Shanghai, China), which were routinely cultured in high-glucose DMEM medium at 37°C with 5% CO_2_.

miR-210 mimics and inhibitors were transfected using Lipofectamine^®^ 2000 (Life Technologies) according to the manufacturer’s protocol. Cells were cultured in 6-well plates until they reached 80% confluence. Subsequently, miR-210 mimics or inhibitors were transfected into the cells using Lipofectamine^®^ 2000 (Invitrogen) at 37°C for 24 h (21). The miR-210 mimic (5′-AGCCACUGCCCACCGCACACUG-3′) and miR-210 inhibitor (5′-CUGUGCGUGUGACAGCGGCUGA-3′) were purchased from MedChemExpress (Anhui, China).

Transfected HK-2, HUVEC, 293T, and NRK-52E cells were cultured in DMEM medium until they adhered. They were then transferred to a hypoxia incubator (1% O_2_, 94% N_2_, 5% CO_2_) and the medium was replaced with D-Hanks solution, which had been aerated with the gas mixture for 10 min (22). For the normoxia model, cells were cultured under standard atmospheric conditions. Additionally, YHF granules were fully dissolved in distilled water and diluted to a concentration of 2 mg/mL. HK-2 and HUVEC cells were treated with YHF at a final concentration of 200 μg/mL for 24 h under normoxic or hypoxic conditions.

### 2.10 Cell proliferation and migration assay

CCK-8 and transwell migration assays were performed to assess cell proliferation and migration. Different groups of cells were separately inoculated in 96-well plates at a density of 2,000 cells per well, and cell proliferation was measured using Cell Counting Kit-8 (Sigma-Aldrich, China) according to the manufacturer’s instructions. Migration assay was performed using a 24-well transwell (Corning, United States). Cells were inoculated in DMEM with no FBS in the upper chamber. the lower chamber was filled with DMEM containing 10% FBS. after 24 h of incubation, the cells were stained using 0.5% crystal violet.

### 2.11 HUVEC tubule formation assay

Each well of a 96-well plate was coated with 50 μL of matrigel, and then HUVEC were inoculated in matrigel-coated 96-well plates at a density of 1 × 10^4^ cells per well. After 24 h of incubation, photographs were taken with a light microscope (23).

### 2.12 Dual-luciferase reporter assay

According to the manufacturer’s protocol of the Dual-Luciferase Reporter Assay Kit (Hanheng Biotechnology, China), firefly luciferase reporter plasmids (wild-type or mutant) were co-transfected with miR-210 mimic or NC mimic into HUVEC cells. After 48 h of transfection, firefly and Renilla luciferase signals were measured using the Dual-Luciferase Reporter Assay Reagent/II and detected with a luminometer (24).

### 2.13 Quantitative real-time PCR (qRT-pCR)

Total RNAs from cells were extracted using TRIzol reagent (Invitrogen, United States) and reverse transcription was performed using a first-strand cDNA synthesis kit (Transgen Biotech, China) according to the manufacturer’s instructions. GAPDH was used as a normalization control for mRNA (25). All primer sequences are listed in Table 1.

**TABLE 1:** Sequences of primers used for Quantitative real-time PCR (qRT-PCR).

Primer	Primer sequence (5′ → 3′)
GAPDH-F	GACAGTCAGCCGCATCTTCT
GAPDH-R	GCCCAATACGACCAAATCCGT
HIF-1α-F	ATCACCCTCTTCGTCGCTTC
HIF-1α-R	GGAAAGGCAAGTCCAGAGGT
BNIP3-F	CACCTCTGCCTGTCCGATTTCAC
BNIP3-R	GAGAGTGCTTGCTGCTTCCATCC
NIX-F	ACAATGTCGTCCCACCTAGTC
NIX-R	TAGCTCCACCCAGGAACTGTTG
U6-F	CTCGCTTCGGCAGCACA
U6-R	AACGCT-TCACGAATTTGCGT
miR-210-F	GTGCAGGGTCCGAGGT
miR-210-R	TATCTGTGCGTGTGACAGCGGCT

### 2.14 Western blot

Total protein was separated from cells by SDS-PAGE and then was transferred to a polyvinylidene difluoride membrane (Millipore, United States). Subsequently, membranes were blocked in TBS with 5% no-fat milk and incubated with primary antibodies overnight at 4°C. After washing with TBST, the membrane was incubated with secondary antibody at 37°C for 1.5 h (26). Protein bands were visualized using ECL Western blotting detection reagent and analyzed for band intensity using ImageJ software. Antibody information are listed in Table 2.

**TABLE 2:** Information of antibody used for Western blot.

Antibody	Producer	Product number
GAPDH	Proteintech	10494-1-AP
HIF-1α	Proteintech	20960-1-AP
BNIP3	Proteintech	68091-1-Ig
BNIP3L/NIX	Proteintech	68118-1-Ig
HRP-conjugated affinipure goat anti-rabbit IgG(H + L)	Proteintech	SA00001-2

### 2.15 Statistical analysis

All data are presented as mean ± standard deviation. Student’s *t* test or one-way analysis of variance (ANOVA) was performed to compare differences among different groups using GraphPad Prism 8. *P*-value < 0.05 indicated a significant statistical difference.

## 3 Results

### 3.1 YHF alleviates RIF in a UUO rat model

**FIGURE 1:**
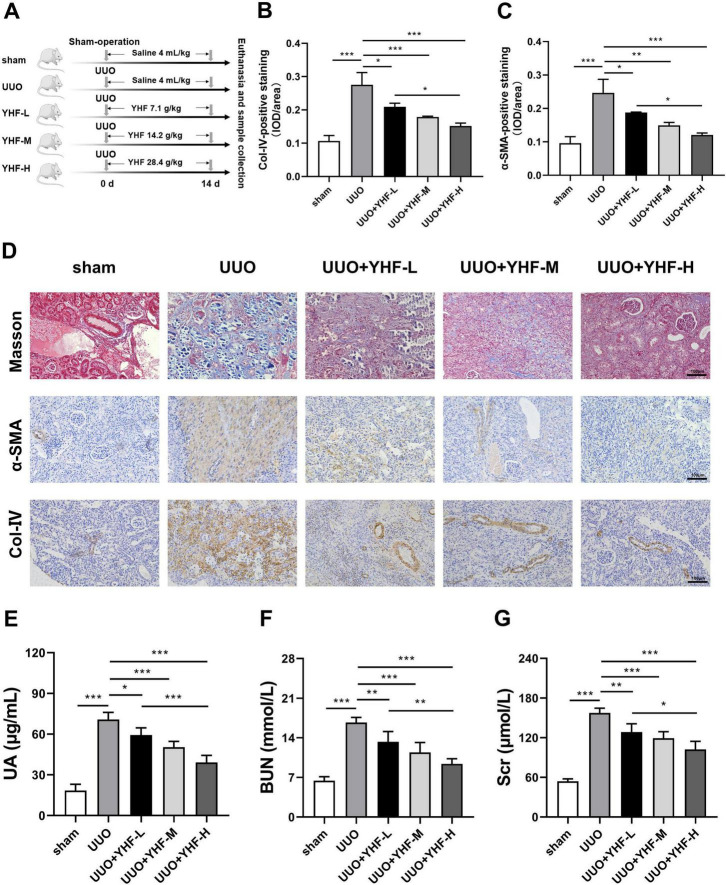
Impact of Yishen-Huoxue formula (YHF) on renal interstitial fibrosis (RIF) in the unilateral ureteral obstruction (UUO) rat model. **(A)** Flowchart of rat treatment procedure. **(B,C)** Quantitative analysis of immunohistochemical staining for Col-IV **(B)** and α-SMA **(C)**. *p < 0.05, **p < 0.01, and ***p < 0.001. **(D)** Masson’s trichrome staining and immunohistochemical staining for α-SMA and Col-IV (magnification × 200). **(E–G)** Indicators of renal function in rats, including uric acid **(E)**, blood urea nitrogen **(F)**, and serum creatinine **(G)**. *p < 0.05, **p < 0.01, and ***p < 0.001.

In this study, an *in vivo* RIF model was successfully induced through UUO. Figure 1A depicts the grouping and handling procedure of the rats. On the 14th day post-surgery, rats were euthanized, and immunohistochemistry was performed to detect the RIF markers, including α-SMA and Col-IV (Figures 1B, C). Compared to the sham group, UUO group exhibited significantly elevated levels of both α-SMA and Col-IV. However, YHF treatment notably counteracted these changes, and the effect of YHF was positively correlated with its dose. This pathological alteration can be more visually observed in the Masson’s trichrome staining and immunohistochemical images shown in Figure 1D. Compared to the sham group, the kidney tissues of rats in the UUO group displayed notably increased collagen fibers (blue), α-SMA (brown), and Col-IV (brown) accumulation, indicating the occurrence of RIF (Figure 1D). Conversely, in the UUO + YHF-L/M/H groups, the degree of RIF in the kidney tissues of UUO rats was significantly mitigated, suggesting that YHF has the effect of alleviating RIF in the UUO rat model.

Furthermore, we assessed the renal function of rats in each group. ELISA results revealed a significant increase in UA levels in the UUO group, whereas YHF treatment significantly reduced UA levels, with a positive correlation to its concentration (Figure 1E). Similarly, serum BUN and Scr expression were elevated in the UUO group, while YHF markedly decreased BUN and Scr levels, also demonstrating a positive correlation with its concentration (Figures 1F, G). These findings indicate that YHF not only alleviates the progression of RIF in UUO rats but also exerts a significant protective effect on their renal function.

### 3.2 miR-210 was screened as a potential target gene for RIF treatment by YHF

Bioinformatics approaches were used to predict potential target genes of YHF in the treatment of RIF. After 14 days of drug treatment, rat serum exosomes were extracted for miRNA sequencing. miRNAs associated with hypoxia/angiogenesis were screened by comparing the exosomal miRNA expression in different groups. As shown in Supplementary Figures 1A–F, there are 12 up-regulated miRNAs and six down-regulated miRNAs in the UUO + YHF-L group compared with the UUO group; 24 up-regulated miRNAs and 13 down-regulated miRNAs in the UUO + Losartan group compared with the UUO group; and seven up-regulated miRNAs and 12 down-regulated miRNAs in the UUO group compared with the blank group. The Supplementary Figures 1G–L shows GO enrichment and KEGG enrichment results of the predicted target genes in different groups. After we overlapped the differential miRNAs of serum exosomes from each group, we obtained four genes: rno-miR-184, rno-miR-210, rno-miR-142, and rno-miR-126a (Supplementary Figure 2). Our previous studies have demonstrated that YHF can ameliorate RIF through the miR-126/VEGF-Notch signaling pathway (10). Given the substantial evidence indicating a close relationship between miR-210 and hypoxia/angiogenesis, we finally chose miR-210 as the gene of interest for the subsequent study (27, 28).

### 3.3 YHF treatment increases miR-210 levels and decreases HIF-1α, BNIP3, and NIX levels in serum exosomes of UUO rats

**FIGURE 2:**
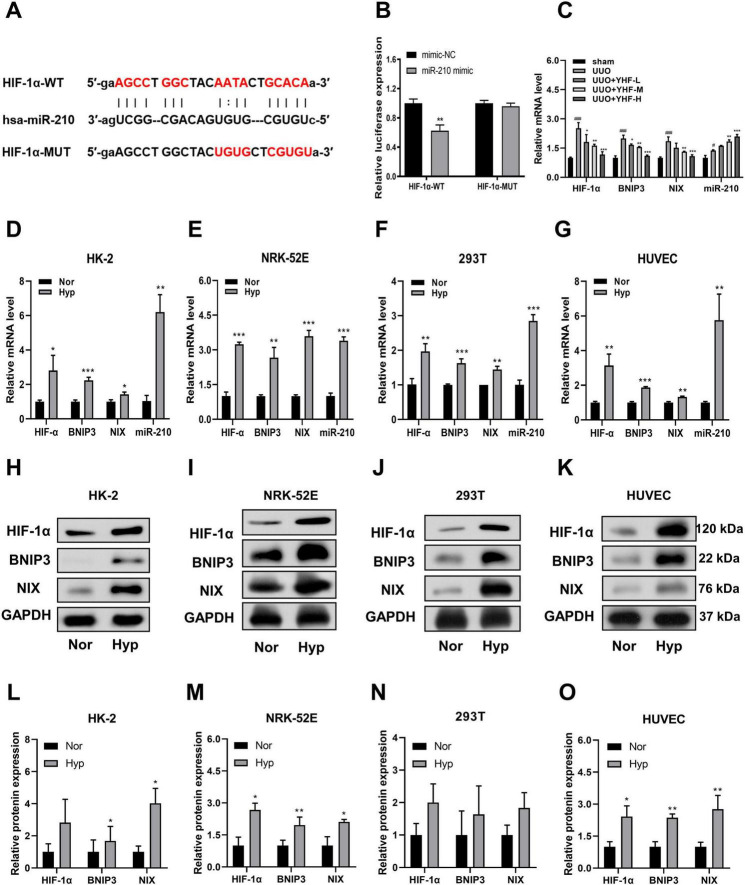
Effects of Yishen-Huoxue formula (YHF) and hypoxia treatment on miR-210/HIF-1α pathway. **(A)** Prediction of the complementary site between HIF-α 3’-untranslated region (3’-UTR) and miR-210 by miRTarBase. **(B)** Bioluminescence reporter assay demonstrating miR-210 targeting of HIF-1α. **p < 0.01. **(C)** Quantitative real-time PCR (qRT-PCR) was performed to detect serum exosomal miR-210 expression and mRNA expression of hypoxia-related factors in rats. #*p* < 0.05, ###*p* < 0.001 vs. sham, and *p < 0.05, **p < 0.01, ***p < 0.001 vs. UUO. **(D–G)** qRT-PCR analysis of miR-210, HIF-1α, BNIP3, and NIX mRNA expression levels in HK-2 **(D)**, NRK-52E **(E)**, 293T **(F)**, and HUVEC **(G)** cells. *p < 0.05, **p < 0.01, and ***p < 0.001. **(H–K)** Western blot analysis of HIF-1α, BNIP3, and NIX expression levels in HK-2 **(H)**, NRK-52E **(I)**, 293T **(J)**, and HUVEC **(K)** cells. **(L–O)** Quantitative analysis of Western blot for HIF-1α, BNIP3, and NIX in HK-2 **(L)**, NRK-52E **(M)**, 293T **(N)**, and HUVEC **(O)** cells. *p < 0.05 and **p < 0.01.

The profibrotic role of HIF-1α has been extensively described in numerous animal models of chronic kidney diseases (29, 30). Furthermore, existing research provides substantial evidence that miR-210 is a robust target of hypoxia-inducible factors, with aberrant expression observed in nearly all types of cancers and ischemic diseases (31). Therefore, we hypothesized that HIF-1α may serve as a pivotal mediator of miR-210 in the mechanism of RIF treatment by YHF. Utilizing miRTarbase, we identified a potential miR-210 binding site within the 3’-untranslated region (3’-UTR) of HIF-1α (Figure 2A). Subsequently, constructs of HIF-1α with either a mutated (HIF-1α-MUT) or wild-type (HIF-1α-WT) 3’-UTR were cloned into a luciferase reporter plasmid. These plasmids were then co-transfected with miR-210 mimics or negative control (NC) mimics into the cells. As shown in Figure 2B, miR-210 mimic significantly decreased luciferase activity in the WT group (*p* < 0.01). In contrast, there was almost no change in luciferase activity in the MUT group, demonstrating that HIF-1α is a direct target of miR-210.

Next, we examined the effects of YHF treatment on the mRNA expression of four hypoxia-related factors in rat serum exosomes using qRT-PCR. As shown in Figure 2C, the mRNA levels of HIF-1α, BNIP3, and NIX were significantly elevated in the UUO groups compared to the sham groups [BNIP3 and NIX have been reported to be downstream genes of HIF-1α and are associated with hypoxia-induced cell death (32, 33)]. Following YHF treatment, the mRNA levels of HIF-1α, BNIP3, and NIX significantly decreased in UUO rats, while the expression of miR-210 increased. This indicated that the expression of factors such as miR-210 and HIF-1α are affected by YHF during RIF. Therefore, the therapeutic effect of YHF on RIF may be related to the miR-210/HIF-1α pathway.

### 3.4 Up-regulation of miR-210 and HIF-1α expression after hypoxia treatment *in vitro*

To verify whether miR-210/HIF-1α pathway plays a role during RIF, an *in vitro* hypoxia model was induced using four cell lines, including human- and rat-derived TECs (HK-2, 293T, and NRK-52E) and ECs (HUVEC). The results of qRT-PCR revealed that the mRNA expression levels of HIF-1α, BNIP3, NIX, and miR-210 were significantly increased in all four cell types following hypoxia treatment (Figures 2D–G). Consistently, Western blotting results suggested that the protein levels of HIF-1α, BNIP3, and NIX were significantly increased or showed an increasing trend after hypoxia treatment (Figures 2H–O).

### 3.5 Hypoxia treatment reduces cell viability and angiogenic capability

**FIGURE 3:**
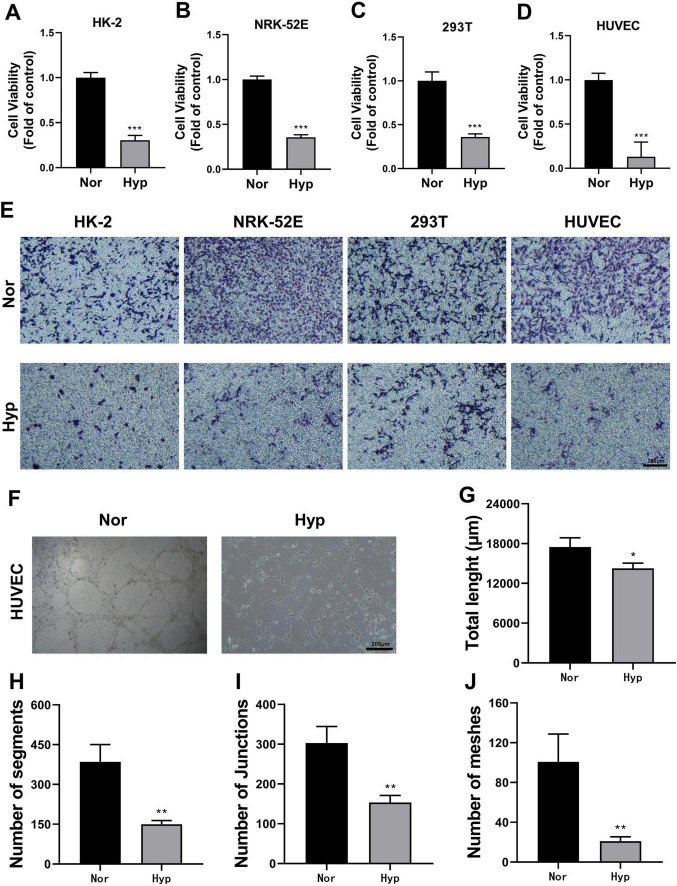
Effect of hypoxia treatment on cell viability and angiogenic capacity. **(A–D)** CCK-8 assays measuring cell proliferation viability of HK-2 **(A)**, NRK-52E **(B)**, 293T **(C)**, and HUVEC **(D)** cells. ***p < 0.001. **(E)** Transwell assay assessing cell migration ability (magnification × 100). **(F)** Tubule formation assay with HUVEC cells (magnification × 100). **(G)** Statistical analysis of total tube length in tubule formation assays. *p <0.05. **(H)** Statistical analysis of segment number in tubule formation assays. **p < 0.01. **(I)** Statistical analysis of junction number in tubule formation assays. **p < 0.01. **(J)** Statistical analysis of mesh number in tubule formation assays. **p < 0.01.

Renal tubules generally have a regenerative ability that contributes to functional recovery after renal injury, which is closely related to the migration, proliferation, and reconstruction of physiological functions of renal tubular cells (34). CCK-8 assays demonstrated a significant decrease in cell proliferation viability in HK-2, HUVEC, 293T, and NRK-52E cells after hypoxic treatment (Figures 3A–D). Transwell assays indicated that hypoxia markedly reduced the migration ability of these cells (Figure 3E). Additionally, tubule formation assays showed that the ability of HUVECs to form tubules was significantly reduced following hypoxia treatment (Figures 3F–J). These results collectively suggested that hypoxia adversely affected cell viability and angiogenic capability in TECs and ECs.

### 3.6 miR-210 overexpression/knockdown affects the expression level of HIF-1α under hypoxic conditions

**FIGURE 4:**
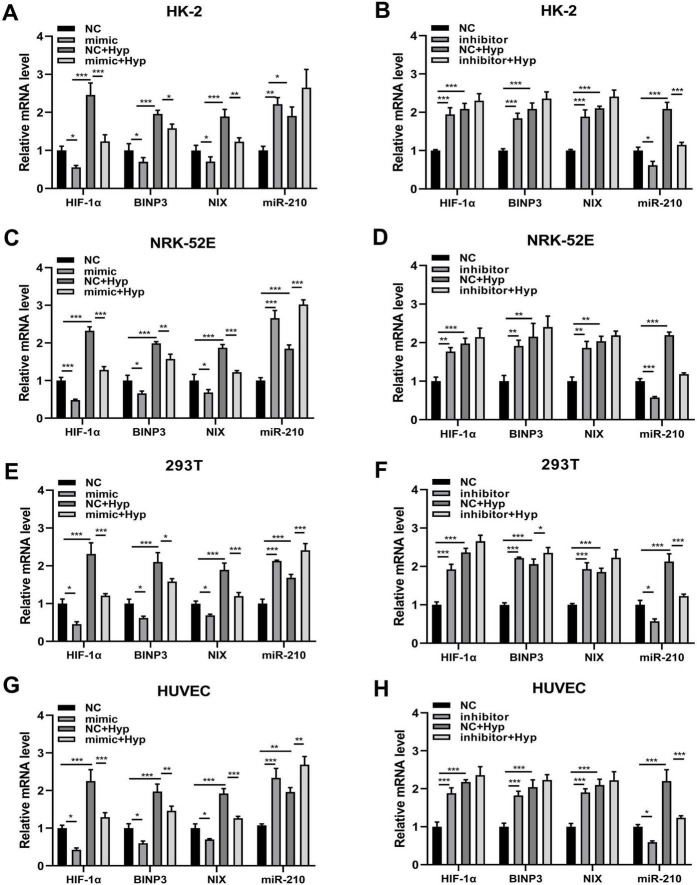
The overexpression/knockdown of miR-210 affects the mRNA expression levels of three factors including HIF-1α, BNIP3, and NIX under hypoxic conditions. **(A–H)** qRT-PCR analysis of mRNA expression levels of HIF-1α, BNIP3, and NIX in cells (HK-2, NRK-52E, 293T, HUVEC) following overexpression **(A,C,E,G)** or knockdown **(B,D,F,H)** of miR-210-3p. *p < 0.05, **p < 0.01, and ***p < 0.001.

As previously described, hypoxia elevated the expression levels of four factors including miR-210 and HIF-1α, where HIF-1α serves as a targeting site for miR-210. Then we further investigated whether miR-210 overexpression or knockdown influences the expression levels of HIF-1α and other factors under hypoxic conditions. qRT-PCR results demonstrated that overexpression of miR-210-3p suppressed the upregulation of HIF-1α, BNIP3, and NIX mRNA expression induced by hypoxia treatment, while knockdown of miR-210 exerted the opposite effect (Figures 4A–H).

### 3.7 miR-210 overexpression/knockdown affects cell viability and angiogenic capability under hypoxic conditions

**FIGURE 5:**
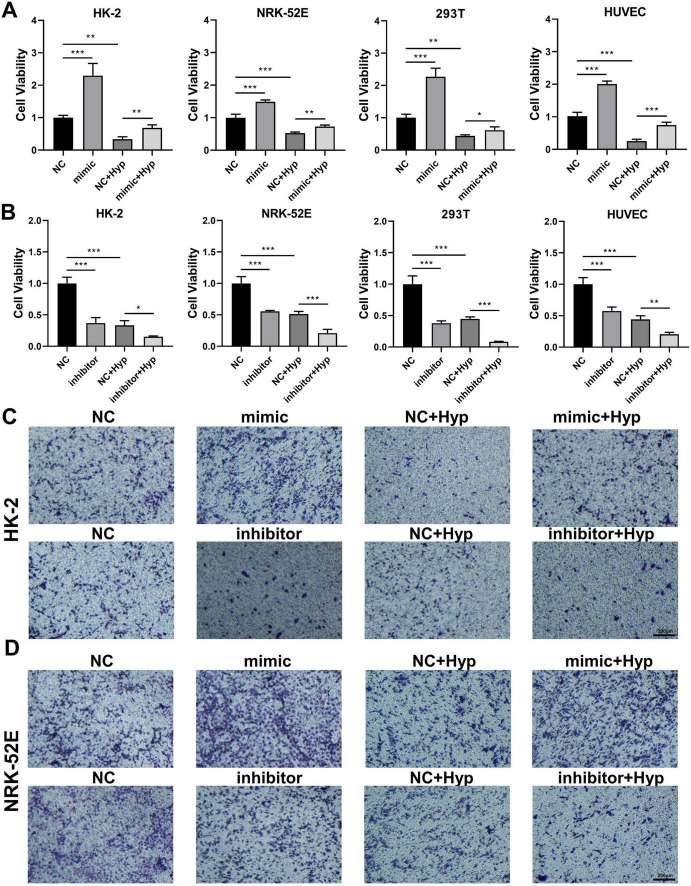
The overexpression/knockdown of miR-210 affects cell viability and migration capacity under hypoxic conditions. **(A,B)** CCK-8 assay measuring cell proliferation viability of HK-2, NRK-52E, 293T, and HUVEC cells following overexpression **(A)** or knockdown **(B)** of miR-210-3p. *p < 0.05, **p < 0.01, and ***p < 0.001. **(C,D)** Transwell assay assessing migration capability of HK-2 **(C)** and NRK-52E **(D)** cells after overexpression or knockdown of miR-210-3p (magnification × 100).

**FIGURE 6:**
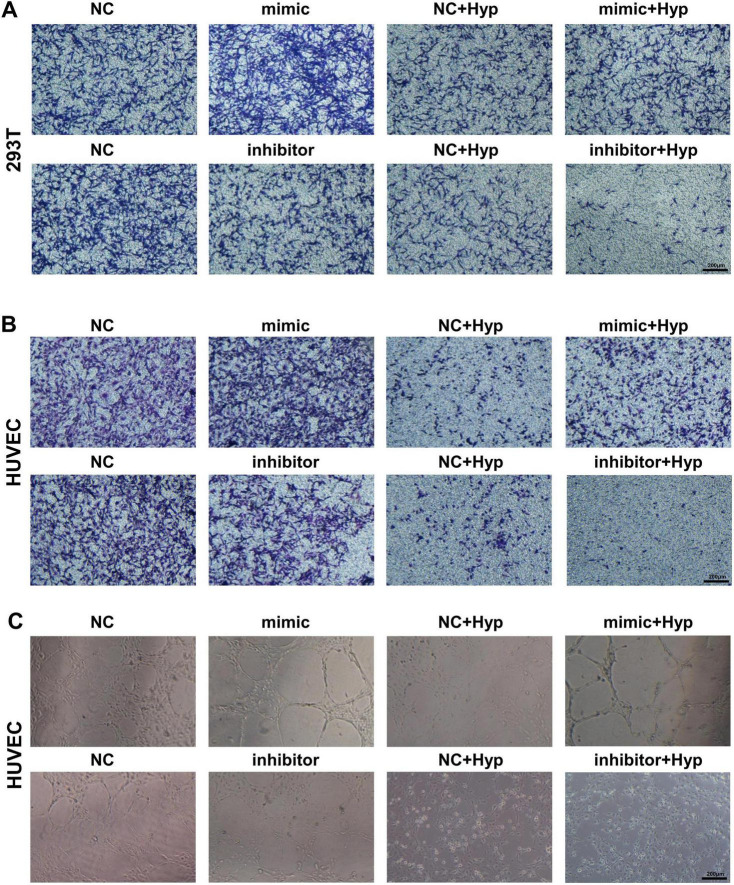
The overexpression/knockdown of miR-210 affects cell migration capacity and angiogenic capacity under hypoxic conditions. **(A,B)** Transwell assay assessing migration capability of 293T **(A)** and HUVEC **(B)** cells after overexpression or knockdown of miR-210-3p (magnification × 100). **(C)** Tubule formation assay assessing angiogenic capability of HUVEC cells after overexpression or knockdown of miR-210-3p (magnification × 100).

Accordingly, we investigated the impact of miR-210 overexpression or knockdown at the cellular level. As depicted in Figure 5A, miR-210 mimics enhanced cell proliferation viability and effectively counteracted part of the decrease induced by hypoxia. On the contrary, miR-210 inhibitor displayed similar effects as hypoxia treatment (Figure 5B). Consistent trends were observed in transwell migration assays (Figures 5C, D, 6A, B), as well as HUVEC tubule formation assays (Figure 6C).

### 3.8 YHF alleviates hypoxia-induced renal cell injury and impaired angiogenesis via the miR-210/HIF-1α pathway

The above results indicate that miR-210 overexpression alleviates hypoxia-induced renal cell injury and impaired angiogenesis by downregulating HIF-1α and related factors. To verify whether YHF exerts its protective effects against RIF through this pathway, we conducted further studies using the following five experimental groups: control, hypoxia, hypoxia + YHF, hypoxia + YHF + miR-210 mimic, and hypoxia + YHF + miR-210 inhibitor groups.

**FIGURE 7:**
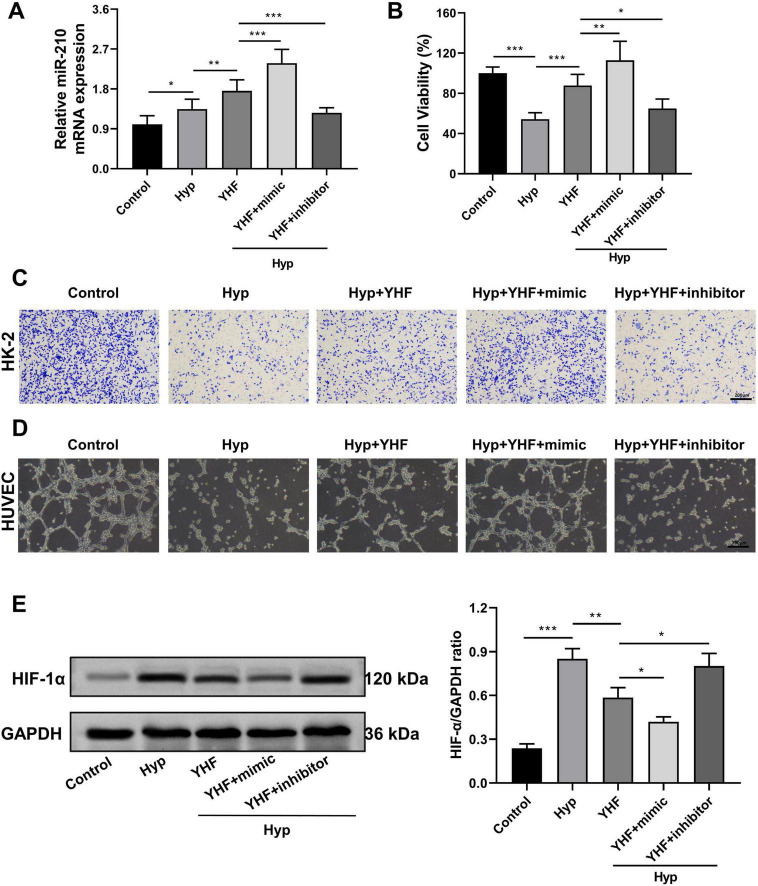
YHF alleviates hypoxia-induced renal cell injury and impaired angiogenesis via the miR-210/HIF-1α pathway. **(A)** qRT-PCR analysis of miR-210 expression levels in HK-2 cells. *p < 0.05, **p < 0.01, and ***p < 0.001. **(B)** CCK-8 assays measuring the proliferation viability of HK-2 cells. **(C)** Transwell assay assessing the migration capability of HK-2 cells (magnification × 100). **(D)** Tubule formation assay of HUVEC (magnification × 100). **(E)** Western blot analysis of HIF-1α expression levels in HK-2 cells. *p < 0.05, **p < 0.01, and ***p < 0.001.

The qRT-PCR results were consistent with *in vivo* findings (Figure 2C) and demonstrated that YHF treatment increased miR-210 expression in HK-2 cells under hypoxic conditions (Figure 7A). Moreover, the CCK-8 assay revealed that YHF treatment mitigated the hypoxia-induced decline in cell viability, an effect that was enhanced by miR-210 mimic and attenuated by miR-210 inhibitor (Figure 7B). Similarly, the transwell migration assays (Figure 7C) and the tubule formation assays (Figure 7D) exhibited the same trend, suggesting that YHF may promote cell viability, migration, and angiogenesis capability under hypoxic conditions by upregulating miR-210.

Subsequently, we assessed HIF-1α protein levels in each group using Western blot analysis. The results showed that YHF treatment inhibited the hypoxia-induced upregulation of HIF-1α in HK-2 cells (Figure 7E). This effect was further enhanced by miR-210 mimic but attenuated by miR-210 inhibitor, indicating that YHF exerts its protective effects against hypoxia-induced renal cell injury and impaired angiogenesis via the miR-210/HIF-1α pathway.

## 4 Discussion

In this study, we investigated the potential mechanisms of the traditional Chinese herbal decoction YHF in alleviating RIF. The results demonstrated that YHF treatment significantly reduced the severity of RIF, mitigated renal pathological damage, and protected renal function in UUO rats. Through miRNA sequencing and differential gene analysis, miR-210 was identified as a potential target gene of YHF in the treatment of RIF. qRT-PCR results confirmed that YHF treatment increased miR-210 levels and decreased HIF-1α, BNIP3, and NIX levels in UUO rats. Subsequently, a series of *in vitro* experiments demonstrated that under hypoxic conditions, miR-210 regulated the levels of HIF-1α and its downstream factors, thereby protecting cellular viability, migration capacity, and angiogenesis. Therefore, the alleviation of renal cellular hypoxic injury and promotion of angiogenesis by YHF through the miR-210/HIF-1α pathway may be one of the underlying mechanisms of its therapeutic effect on RIF.

Yishen-Huoxue formula is a formulation based on traditional Chinese medicine (TCM) theory, composed of six herbs specifically selected for their roles in tonifying the kidney, promoting blood circulation, replenishing Qi, and unblocking the meridians according to TCM principles. Our previous work has observed the significant anti-RIF effects of YHF in the UUO rat model by comparing its efficacy with other renoprotective drugs like chlorosartan (10). However, the mechanism of YHF in RIF treatment is not fully understood. In the present study, we identified four candidates for therapeutic targets, including miR-210, by sequencing and differential analysis of miRNAs in serum exosomes of rats. qRT-PCR results further demonstrated that YHF treatment elevated serum miR-210 levels and reduced the levels of hypoxia-related factors such as HIF-1α in UUO rats, suggesting that miR-210 may serve as a crucial target for YHF in alleviating RIF.

miR-210 is encoded by MIR210 gene on chromosome 11p15.5, with imperative effects in the pathophysiology of human disorders (35). This miRNA represents the major hypoxia-inducible miRNA and affects many key cellular processes, including angiogenesis, metastasis and apoptosis (36). However, the role of miR-210 in hypoxic renal injury has been relatively less explored. In this study, we first demonstrated through a luciferase reporter assay that HIF-1α is a target of miR-210, which can target the 3’ UTR of HIF-1α mRNA. Subsequently, in an *in vitro* hypoxia model, we observed that hypoxia induced an elevation in the levels of miR-210, HIF-1α, BNIP3, and NIX. Additionally, hypoxia resulted in decreased cell viability and migration capacity in human- and rat-derived TECs, as well as reduced tubule-forming ability in HUVECs. Our findings suggested that hypoxia-induced renal tubular cell injury and vascular endothelial damage are associated with increased levels of HIF-1α and its downstream factors. This is consistent with the study by Zhang et al. (37), who also identified HIF-1α as a key mediator of reduced angiogenesis in RIF induced by hypoxia. These hypoxic injuries are detrimental to renal microvascular neogenesis, which can ultimately lead to PTC loss and exacerbation of RIF (38).

*In vivo* experiments demonstrated that YHF treatment increased serum miR-210 levels while decreasing HIF-1α levels in UUO rats. Then, we performed miR-210 overexpression and knockdown experiments in four cell lines to further validate the role of the miR-210/HIF-1α pathway in alleviating RIF. As anticipated, overexpression of miR-210 significantly inhibited the HIF-1α pathway, leading to a reduction in hypoxia-induced cell damage, including decreased cell viability, impaired cell migration, and reduced tubule-forming ability. Conversely, knockdown of miR-210 exhibited the opposite effect. The findings of Liu et al. also verified that miR-210 is involved in the molecular response in hypoxic kidney lesions *in vivo* and attenuates hypoxia-induced renal tubular cell apoptosis by targeting HIF-1α directly and suppressing HIF-1α pathway activation *in vitro* (12). Therefore, the miR-210/HIF-1α pathway may be a potential therapeutic target for the treatment of hypoxic kidney injury. Finally, we repeated the experiments using HK-2 and HUVEC cells treated with YHF. Aligned with the *in vivo* findings, YHF treatment increased miR-210 expression while reducing HIF-1α protein levels. Moreover, YHF treatment alleviated hypoxia-induced renal cell damage and impaired angiogenesis. This protective effect was further enhanced by miR-210 overexpression and attenuated by miR-210 knockdown. Collectively, both *in vivo* and *in vitro* experiments confirmed that YHF mitigates RIF through the miR-210/HIF-1α pathway.

Notably, network pharmacology analysis suggests that, in addition to HIF-1α signaling, the therapeutic mechanisms of YHF may also involve the VEGF and TNF signaling pathways (39). As mentioned above, our prior research has demonstrated that YHF promotes renal microvascular angiogenesis and alleviates microcirculatory damage by upregulating the miR-126/VEGF-Notch signaling pathway (10). Furthermore, the TGF-β/Smad signaling pathway, a key regulatory mechanism in fibrosis, has also been found to be closely associated with YHF’s effects (40). These pathways may act in coordination with the miR-210/HIF-1α axis to regulate RIF. Besides, the roles of the Wnt/β-catenin (41), MAPK (42), and PI3K/AKT (43) signaling pathways in RIF have been widely reported, but whether they also contribute to the anti-RIF effects of YHF remains to be explored. Future studies integrating multi-omics data and experimental validation are expected to provide a more comprehensive understanding of YHF’s molecular network in RIF treatment.

## 5 Conclusion

This study demonstrates that YHF exerts its anti-RIF effects by mediating the miR-210/HIF-1α signaling pathway, increasing cell viability, migration, and promoting angiogenesis. This research not only provides important experimental evidence for the mechanism of the miR-210/HIF-1α pathway in hypoxic kidney disease, but also holds significant implications for the selection of effective traditional Chinese medicine formulas or monomeric anti-RIF.

## Data Availability

The original contributions presented in this study are included in this article/Supplementary material, further inquiries can be directed to the corresponding author.

## Footnotes

1
http://www.cytoscape.org/

2
http://www.geneontology.org/

3
https://david.ncifcrf.gov/

## Supplementary material

Supplementary Figure 1 Differential gene expression and functional enrichment analysis of miRNA in serum exosomes of rats. **(A–C)** Volcano plot showing the differential miRNA in UUO + YHF-L (A), UUO + Losartan **(B)**, and blank **(C)** group compared to unilateral ureteral obstruction (UUO) group. **(D–F)** Heatmap showing miRNA expression differences in UUO + YHF-L **(D)**, UUO + Losartan **(E)**, and blank **(F)** groups compared to UUO group. **(G–I)** Gene Ontology (GO) enrichment analysis of differential genes. **(J–L)** KEGG enrichment analysis of differential genes.

Supplementary Figure 2 Overlap of differential genes between groups.
