# Supplementary figures and images for: Yishen-Huoxue formula alleviates renal interstitial fibrosis by attenuating hypoxia-induced renal cell injury and promoting angiogenesis via miR-210/HIF-1α pathway
Source: Front Med (Lausanne). 2025 May 21;10:1530092. doi: 10.3389/fmed.2025.1530092 (PMC12133958; doi:10.3389/fmed.2025.1530092)

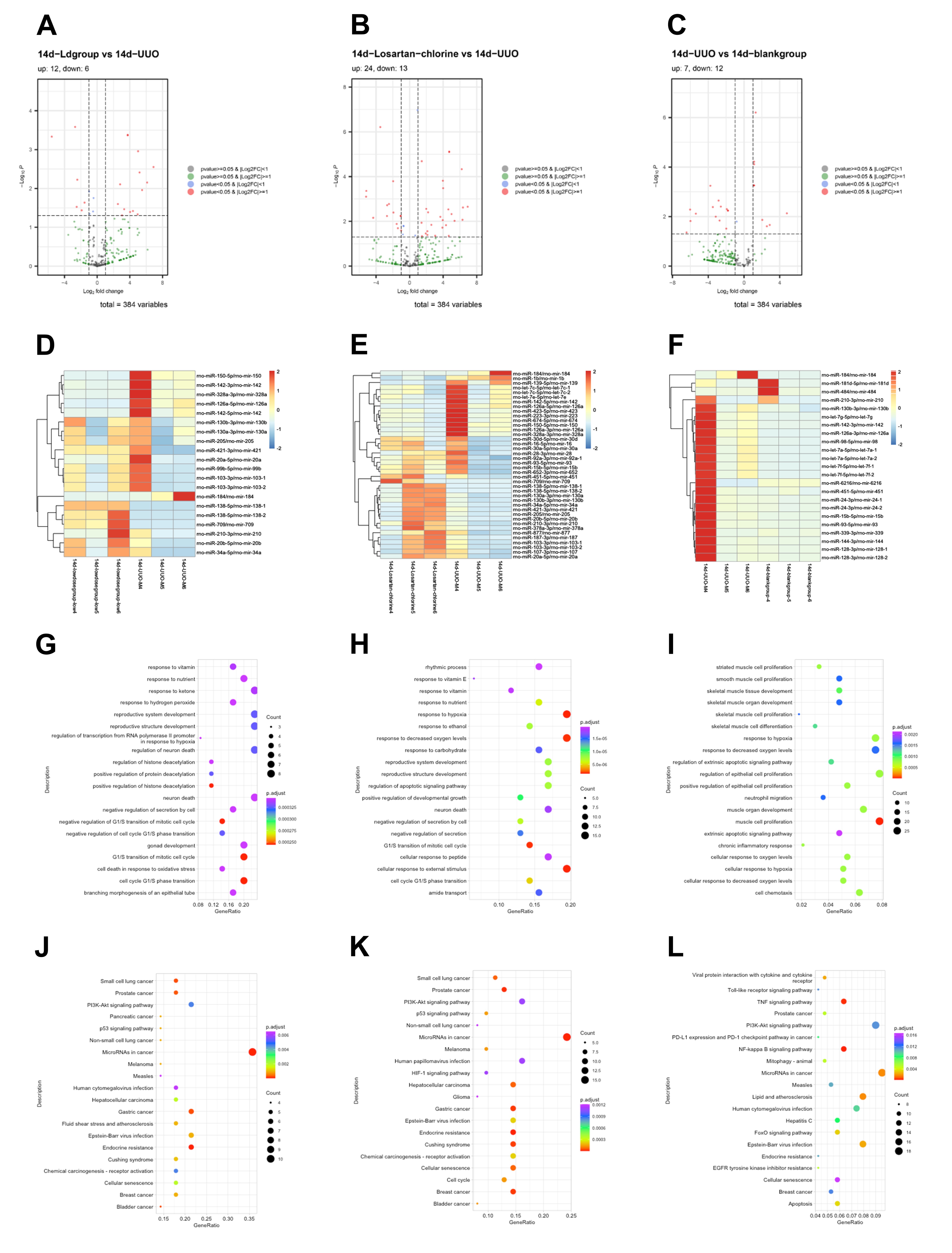

Supplement: Supplementary Figure 1 — Differential gene expression and functional enrichment analysis of miRNA in serum exosomes of rats. (A–C) Volcano plot showing the differential miRNA in UUO + YHF-L (A), UUO + Losartan (B), and blank (C) group compared to unilateral ureteral obstruction (UUO) group. (D–F) Heatmap showing miRNA expression differences in UUO + YHF-L (D), UUO + Losartan (E), and blank (F) groups compared to UUO group. (G–I) Gene Ontology (GO) enrichment analysis of differential genes. (J–L) KEGG enrichment analysis of differential genes. [file Image_1.jpeg]

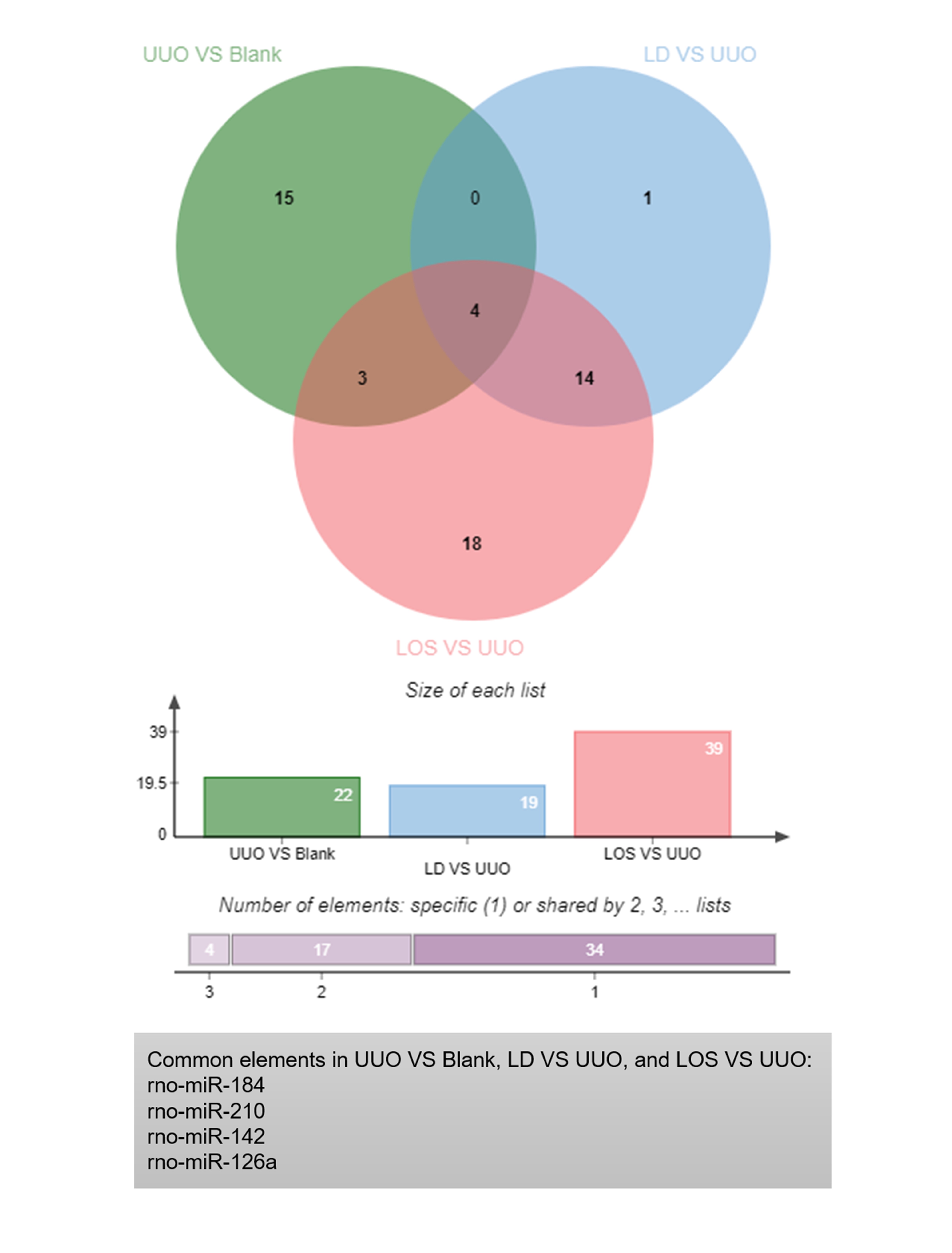

Supplement: Supplementary Figure 2 — Overlap of differential genes between groups. [file Image_2.jpeg]
